# Liver stiffness and insulin resistance in predicting recurrence for early stage hepatoma patients after curative resection

**DOI:** 10.1038/s41598-021-85431-y

**Published:** 2021-03-15

**Authors:** Jing-Houng Wang, Wei-Feng Li, Chee-Chien Yong, Yueh-Wei Liu, Sheng-Nan Lu, Chih-Chi Wang

**Affiliations:** 1 Department of Internal Medicine, Kaohsiung Chang Gung Memorial Hospital and Chang Gung University College of Medicine, Kaohsiung City, Taiwan; 2 Department of Surgery, Kaohsiung Chang Gung Memorial Hospital and Chang Gung University College of Medicine, 123, Ta Pei Road, Niao Sung 833, Kaohsiung City, Taiwan

**Keywords:** Gastroenterology, Oncology, Risk factors

## Abstract

Curative resection is recommended for patient with early stage hepatocellular carcinoma (HCC), however, the prognosis is limited by high recurrence rate. This study was to investigate liver stiffness (LS) and metabolic factor in prediction of HCC recurrence for patients with early stage HCC who had undergone curative resection. Consecutive patients with suspicion of HCC who had undergone curative resection were prospectively enrolled. Transient elastography was performed to determine LS pre-operatively. The demographics, clinical characteristics and histological findings were recorded. All patients were followed up regularly until recurrence, death or last visit. Ninety-four patients with early stage HCC were enrolled. LS positively correlated with fibrosis stage (r = 0.666). In a median follow-up of 3.2 years, forty patients developed recurrences including 22 recurrences after 1-year post resection. The 5-year cumulative recurrence rate was 44.2%. LS was the independent factor associated with recurrence. Patients with LS > 8.5 kPa had higher 5-year cumulative recurrence rate (59.8% vs 25.1%, p = 0.007). For the prediction of recurrence after 1-year post resection, LS > 8.5 kPa (hazard ratio 2.72) and homeostatic model assessment for insulin resistance index (HOMA-IR) (hazard ratio 1.24) were independent factors in multivariate analysis. Those patients with both LS > 8.5 kPa and HOMA-IR > 2.3 had the highest recurrence rate after 1-year post resection.

## Introduction

Liver cancer is among the leading causes of cancer death with more than eight hundred thousand deaths reported globally in 2015^1^. For high-risk patients, hepatocellular carcinoma (HCC) surveillance is recommended to detect small HCC where curative treatments will improve survival^2,3^. Among curative treatments, surgical resection is recommended by guidelines among the first-line treatment options for early-stage HCC patient with well liver function reserve and good performance status^4–6^. However, the recurrence rate was more than 50% at 5 years and resulted in a 24% decrease in the 5-year survival rate and a 54-month decrease in median survival^7,8^. It is important to identify and follow those patients at high risk of recurrence, for whom early recurrence predicted poor prognosis and curative intent was recommended because of high rates of treatable recurrence^4,8,9^.

The factors associated with recurrence included tumor characteristics, surgical techniques, background stage of liver disease and hepatitis activity^7,10–12^. Hepatic fibrosis was associated with not only high recurrence rate, but recurrence pattern as well, which might affect survival after recurrence^7,9,12^. Liver stiffness measurement with transient elastography has been demonstrated to be in good correlation with the extent of hepatic fibrosis and be a reliable method for the diagnosis of cirrhosis in patients with chronic liver disease. It is recommended as the non-invasive standard for LS measurement in the assessment of hepatic fibrosis and useful to identify patients at risk of disease progression including HCC development^13^. For patients with HCC undergoing curative treatments including resection and radiofrequency ablation, pre-therapy LS measurement using transient elastography was demonstrated to be useful in risk stratification of recurrence after treatments in Asian patients^14,15^.

However, LS was not the independent factor associated with HCC recurrence in recent reports from western countries^16,17^. The inconsistent results might be due to heterogenous HCC stage in patient populations at the enrollment. To further clarify the issue, it is important to enroll a homogenous stage of HCC patients to delineate the usefulness of LS in recurrence prediction. In addition, metabolic factor might be the independent factor of HCC recurrence which was not assessed in previous studies^14,16^. Therefore, the purpose of this study was to investigate whether LS and metabolic factor were useful in predicting tumor recurrence for patients with early stage HCC after curative resection.

## Results

### Patients

**Figure 1:**
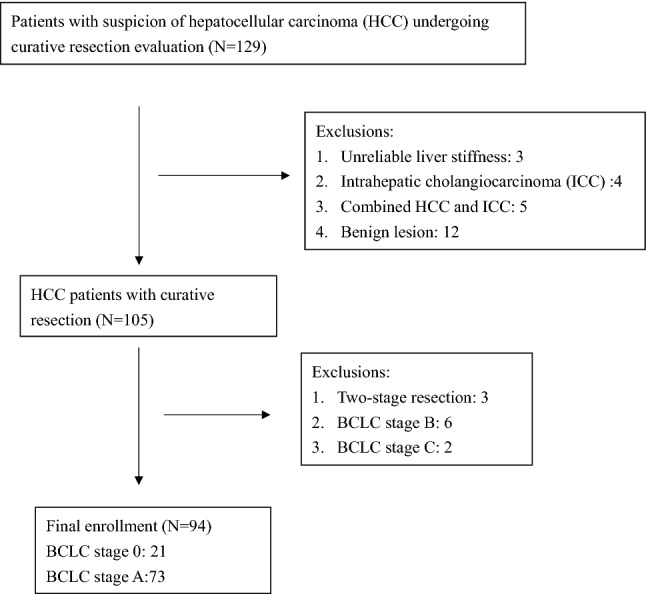
The flow chart for the enrollment of patients with hepatocellular carcinoma (HCC) in Barcelona clinic liver cancer (BCLC) stages 0 and A.

**Figure 2:**
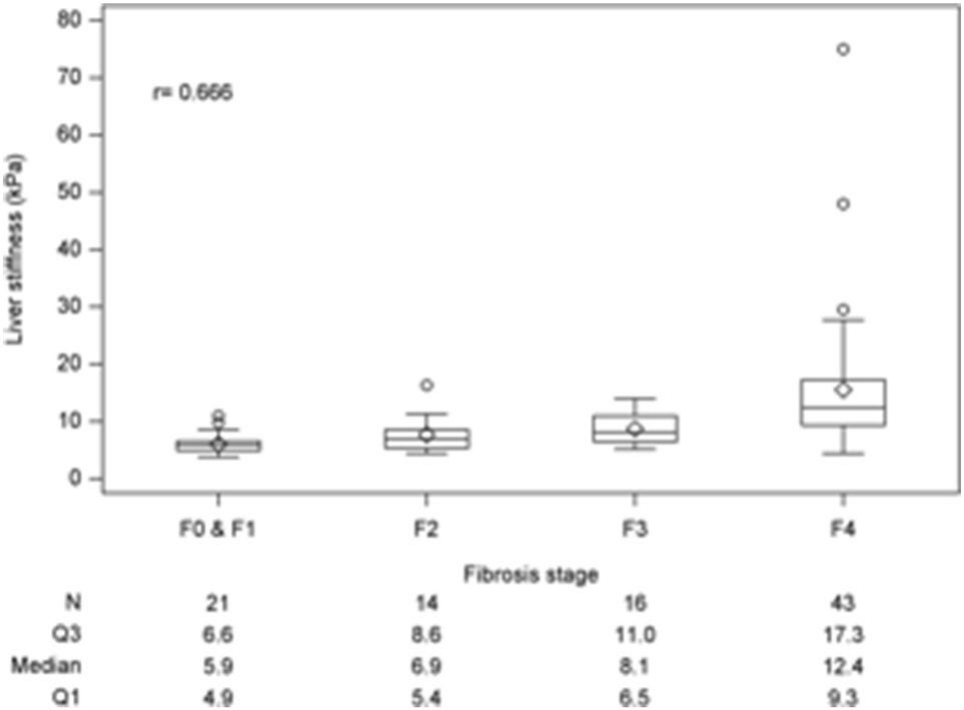
Liver stiffness correlated with histological fibrosis stage (F) positively (r = 0.666).

Between August 2012 and February 2016, 129 patients underwent curative resection of liver tumor with suspicion of HCC. A total of 94 patients (male/female 74/20) with a median age of 62.2 years were enrolled to this study after excluding 3 patients with unreliable LS, 21 with non-HCC diagnosis, 8 in Barcelona Clinic Liver Cancer (BCLC) stage B/C , and 3 patients with two-stage surgical resection (Fig. 1). The median LS was 8.6 kPa and tumor diameter was 2.5 cm. There were 44 patients with hepatitis B virus-related HCC, 73 in BCLC stage A, 59 with fibrosis stage 3/4, and 44 with microvascular invasion (Table 1). Sixty patients underwent antiviral therapy including 34 HBV with oral nucleotide agents in complete virological response and 26 hepatitis C virus infection with interferon-based therapy in sustained virological response. LS was positively correlated with histological fibrosis stage (F) (Fig. 2). The median LS value was 5.9 kPa, 6.9 kPa, 8.1 kPa and 12.4 kPa for F0/1, F2, F3 and F4, respectively.

**Table 1:** The demographics and baseline characteristics of all enrolled patients, patients with and without tumor recurrence (rec).

Characteristics	All (n = 94)	Rec (n = 40)	No rec (n = 54)	p*
Age (year, median, range)	62.2 (33.8–83.5)	63.9 (39.9–83.3)	59.8 (33.8–83.5)	0.165
**Sex (%)**				0.795
Female	20 (21.3)	8 (20.0)	12 (22.2)	
Male	74 (78.7)	32 (80.0)	42 (77.8)	
**Hepatitis etiology (%)**				0.124
NBNC	7 (7.4)	1 (2.5)	6 (11.1)	
B	44 (46.8 )	16 (40.0)	28 (51.9)	
C	39 (41.5)	20 (50.0)	19 (35.2)	
B + C	4 (4.3)	3 (7.5)	1 (1.9)	
**Diabetes mellitus (%)**				0.974
No	73 (77.7)	31 (77.5)	42 (77.8)	
Yes	21 (22.3)	9 (22.5)	12 (22.2)	
BMI (kg/m^2^, median, range)	25.1 (17.4–45.7)	25.2 (17.4–45.7)	24.8 (18.6–40.6)	0.851
Waist (cm, median, range)	88.5 (68.0–108.0)	90.0 (71.0–108.0)	87.0 (68.0–108.0)	0.587
**Metabolic syndrome (%)**				0.272
No	60 (63.8)	23 (57.5)	37 (68.5)	
Yes	34 (36.2)	17 (42.5	17 (31.5)	
Tumor (cm, median, range)	2.5 (0.7–5.0)	2.7 (0.7–5.0)	2.4 (1.0–5.0)	0.555
**Tumor number (%)**				1.000
Single	86 (91.5)	37 (92.5)	49 (90.7)	
Two	8 (8.5)	3 (7.5)	5 (9.3)	
**Fibrosis stage (%)**				0.212
0/1/2	35 (37.2)	12 (30.0)	23 (42.6)	
3/4	59 (62.8)	28 (70.0)	31 (57.4)	
**Histology grade of HCC (%)**				0.489
I	12 (12.8)	4 (10.0)	8 (14.8)	
II	82 (87.2)	36 (90.0)	46 (85.2)	
**Microvascular invasion (%)**				0.341
No	50 (53.2)	19 (47.5)	31 (57.4)	
Yes	44 (46.8)	21 (52.5)	23 (42.6)	
**BCLC stage (%)**				0.974
0	21 (22.3)	9 (22.5)	12 (22.2)	
A	73 (77.7)	31 (77.5)	42 (77.8)	
**Antiviral treatment (%)**				0.132
Yes	60 (63.8)	29 (72.5)	31 (57.4)	
No	34 (36.2)	11 (27.5)	23 (42.6)	
**ALBI grade (%)**				0.648
I	89 (94.7)	37 (92.5)	52 (96.3)	
II	5 (5.3)	3 (7.5)	2 (3.7)	
*ALB *(g/dL, median, range)	4.2 (3.3–5.0)	4.2 (3.3–4.8)	4.3 (3.5–5.0)	0.651
BIL (mg/dL, median, range)	0.7 (0.2–1.4)	0.7 (0.3–1.4)	0.7 (0.2–1.4)	0.550
AST (U/L, median, range)	34.0 (12.0–131.0)	32.0 (12.0–131.0)	34.0 (18.0–93.0)	0.988
ALT (U/L, median, range)	37.5 (13.0–174.0)	36.5 (13.0–174.0)	37.5 (13.0–141.0)	0.463
GGT (U/L, median, range)	19.0 (6.9–121.0)	19.0 (9.0–77.0)	19.0 (6.9–121.0)	0.984
ALP (U/L, median, range)	64.0 (32.0–143.0)	70.0 (39.0–143.0)	62.0 (32.0–119.0)	0.076
**Platelets (× 10**^**3**^**/μL, %)**				0.801
≥ 150	55 (58.5)	24 (60.0)	31 (57.4)	
< 150	39 (41.5)	16 (40.0)	23 (42.6)	
**AFP (ng/mL, %)**				0.667
< 15	54 (57.4)	24 (60.0)	30 (55.6)	
≥ 15	40 (42.6)	16 (40.0)	24 (44.4)	
*HDL* (mg/dL, median, range)	47.5 (26.0–111.0)	47.0 (26.0–81.0)	47.5 (27.0–111.0)	0.472
LDL (mg/dL, median, range)	92.0 (13.0–160.0)	92.5 (18.0–144.0)	92.0 (13.0–160.0)	0.972
TG (mg/dL, median, range)	87.0 (36.0–428.0)	90.5 (36.0–227.0)	82.5 (41.0–428.0)	0.155
LS (kPa, median, range)	8.6 (3.8–75.0)	10.3 (3.8–75.0)	7.0 (3.8–26.3)	0.020
ICG retention rate (%)	7.6 (1.1–39.2)	9.2 (2.2–39.2)	7.0 (1.1–35.0)	0.042
MELD score (median, range)	7.0 (6.4–11.1)	7.2 (6.4–11.0)	6.9 (6.4–11.1)	0.234
HOMA-IR (median, range)	1.3 (0.2–16.2)	1.0 (0.3–16.2)	1.3 (0.2–7.5)	0.566

*B* hepatitis B virus, *C* hepatitis C virus, *NBNC* non-hepatitis B virus and non-hepatitis C virus, *BMI* body mass index, *BCLC* Barcelona clinic liver cancer, *LS* liver stiffness, *ICG* indocyanine green, *HDL* high density lipoprotein, *LDL* low density lipoprotein, *TG* triglyceride, *ALB* albumin, *BIL* total bilirubin, *AST* aspartate aminotransferase, *ALT* alanine aminotransferase, *GGT* gamma glutamyl transferase, *ALP* alkaline phosphatase, *AFP* alpha-fetoprotein, *AL*BI albumin-bilirubin grade, *MELD* model for end stage liver disease, *HOMA-IR* homeostatic model assessment for insulin resistance, p* comparisons between groups with and without recurrence.

### LS in prediction of HCC recurrence

**Figure 3:**
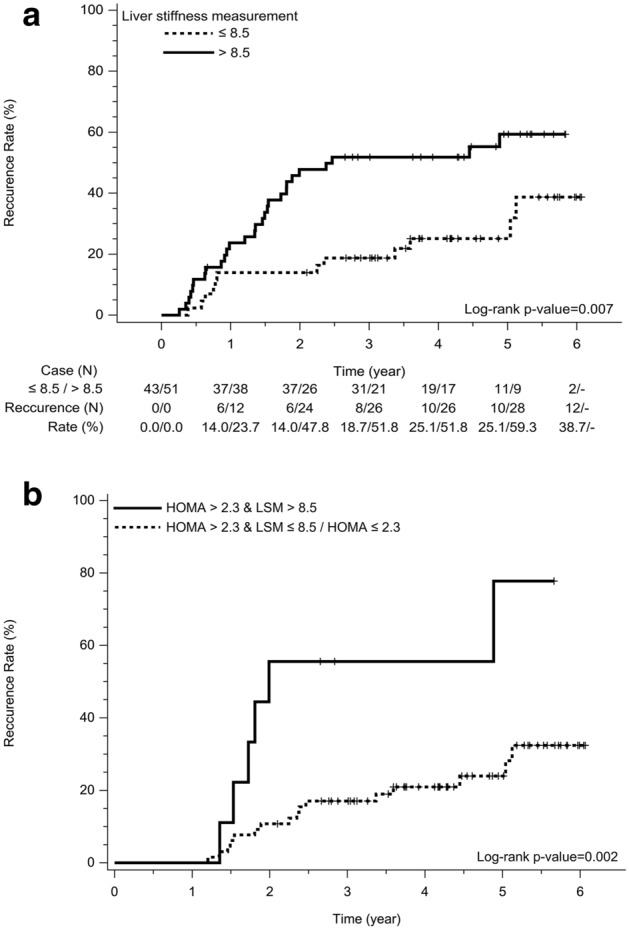
Stratified by 8.5 kPa in liver stiffness (LS) and 2.3 in homeostatic model assessment for insulin resistance index (HOMA), a. those patients with LS > 8.5 kPa had a significantly higher recurrence rate and b. those patients with both LS > 8.5 kPa and HOMA > 2.3 had the highest recurrence rate after 1-year post resection.

There were 40 patients with HCC recurrence, including 39 patients with intrahepatic locations and one patient with only extra-hepatic locations, in a median follow-up of 3.2 years (range 0.26–6.07). Thirty-two patients had solitary recurrence, with the recurrence patterns being BCLC stage 0 in 20 patients, A in 14 patients, B in 4 patients and C in 2 patients. The one-, 3- and 5-year cumulated recurrence rates were 19.2%, 36.5% and 44.2%, respectively. Multi-variate analysis showed LS was the independent factor associated with tumor recurrence (HR 1.03, 95% CI 1.01–1.05; p = 0.011) (Table 2). The performance in prediction of tumor recurrence was 0.641 assessed with AUROC. With the cutoff of 8.5 kPa, the sensitivity, specificity, accuracy, positive and negative predictive values were 70%, 57.4%, 62.8%, 54.9% and 72.1% respectively. For the 43 patients with LS ≤ 8.5 kPa, the 1-, 3- and 5-year cumulative recurrence rates were 14%, 18.7% and 25.1%, which were lower than 23.7%, 51.8% and 59.3% for those with LS > 8.5 kPa (p = 0.007) (Fig. 3a).

**Table 2:** Uni- and multi-variate analysis of factors associated with hepatoma recurrence (n = 94).

Associated factor	Comparison	Univariate HR (95% CI)	p value	Multivariate* HR (95% CI)	p value
Hepatitis etiology	NBC	Reference			
	B	2.86 (0.38–21.57)	0.308		
	C	4.73 (0.63–35.32)	0.129		
	BC	11.52 (1.18–112.03)	0.035		
Hepatitis treatment	Yes No	Reference 0.56 (0.28–1.13)	0.105		
LS (kPa)		1.03 (1.01–1.05)	0.014	1.03 (1.01–1.05)	0.011
ICG (%)		1.04 (1.00–1.08)	0.053		
AST (U/L)		1.01 (1.00–1.02)	0.087		
ALP (U/L)		1.02 (1.00–1.03)	0.035		
MELD		1.22 (0.94–1.58)	0.127		
HOMA-IR		1.11 (1.00–1.23)	0.057		

*B* hepatitis B virus, *C* hepatitis C virus, *NBNC* non-hepatitis B virus and non-hepatitis C virus, *LS* liver stiffness, *ICG* indocyanine green, *AST* aspartate aminotransferase, *ALP* alkaline phosphatase, *MELD* model for end stage liver disease, *HOMA-IR* homeostatic model assessment for insulin resistance, *all characteristics in Table 1 were included in the univariable analysis and those with p values < 0.2 was entered into stepwise multivariable analysis.

### HCC recurrence after 1-year post resection

After excluding 18 patients with recurrence within 1 year post resection and one patient without complete data, there were 75 patients with more than 1 year of follow-up. Among these 75 patients, there were 22 patients with recurrence after curative resections. Multivariate analysis showed LS > 8.5 kPa (HR 2.72, 95% CI 1.05–7.07; p = 0.039) and HOMA-IR (HR 1.24, 95% CI 1.07–1.44; p = 0.003) were independently associated with recurrence (Table 3). The performance of HOMA-IR was 0.553 in recurrence prediction and the optimal cutoff was 2.3 by ROC curve. With the cutoffs of 8.5 kPa and 2.3 for LS and HOMA-IR, patients with both LS and HOMA-IR more than the cutoffs had higher recurrence than those without (p = 0.002) (Fig. 3b).

**Table 3:** Uni- and multi-variate analysis of factors associated with late recurrence (n = 75).

Model 1					
Associated factor	Comparison	Univariate HR (95% CI)	p value	Multivariate* HR (95% CI)	p value
Hepatitis etiology	NBC	Reference			
	B	1.82 (0.23–14.25)	0.568		
	C	2.64 (0.34–20.67)	0.355		
	BC	6.55 (0.40–107.64)	0.188		
Fibrosis stage	0/1/2	Reference			
	3/4	1.88 (0.74–4.81)	0.187		
LS (kPa)	≤ 8.5	Reference		Reference	
	> 8.5	3.18 (1.24–8.14)	0.016	2.72 (1.05–7.07)	0.039
ICG (%)		1.04 (0.99–1.09)	0.152		
ALP (U/L)		1.03 (1.01–1.04)	0.008		
HOMA-IR		1.25 (1.08–1.44)	0.003	1.24 (1.07–1.44)	0.003
**Model 2**					
Hepatitis etiology	NBC	Reference	0.568		
	B	1.82 (0.23–14.25)			
	C	2.64 (0.34–20.67)	0.355		
	BC	6.55 (0.40–107.64)	0.188		
Fibrosis stage	0/1/2	Reference	0.187		
	3/4	1.88 (0.74–4.81)			
ICG (%)		1.04 (0.99–1.09)	0.152		
ALP (U/L)		1.03 (1.01–1.04)	0.008		
HOMA-IR and LS	HOMA ≤ 2.3 and LS ≤ 8.5	Reference		Reference	
	HOMA > 2.3 and LS ≤ 8.5	1.11 (0.13–9.57)	0.921	1.09 (0.13–9.39)	0.935
	HOMA ≤ 2.3 and LS > 8.5	2.42 (0.83–7.10)	0.106	2.15 (0.72–6.41)	0.171
	HOMA > 2.3 and LS > 8.5	6.50 (1.97–21.52)	0.002	6.47(1.95–21.42)	0.002

*B* hepatitis B virus, *C* hepatitis C virus, *NBNC* non-hepatitis B virus and non-hepatitis C virus, *LS* liver stiffness, *ICG* indocyanine green, *ALP* alkaline phosphatase, *HOMA-IR* homeostatic model assessment for insulin resistance, *all characteristics in Table 1 were included in the univariable analysis and those with p values < 0.2 were entered into stepwise multivariable analysis.

## Discussion

The risk of HCC recurrence after resection was bimodal with higher incidence within 1-year follow-up after resection^12,18^. In this prospective study, we demonstrated that LS by transient elastography was in correlation with fibrosis stage and useful in the prediction of all and recurrence after 1-year post resection in a cohort of 94 patients in BCLC early stage HCC. However, the performance and prediction validities were not satisfactory. Patients with LS > 8.5 kPa had a higher 5-year cumulative recurrence rate than those without. In addition to LS, HOMA-IR was independently associated with recurrence after 1-year post resection. There were higher late recurrence rates for those patients with LS > 8.5 kPa and HOMA-IR > 2.3.

The 5-year cumulative HCC recurrence rate was 50%-70% with intrahepatic locations in up to 66–70% after resection^8,10^. In this study cohort, the 5-year HCC recurrence rate was 44.2%, which was lower than that reported in the literature^10^. The proportions of patients with recurrence at intrahepatic location and in BCLC early stage pattern were 97.5% and 85%, which were higher than those reported in other studies^7,8,10^. The enrollment of an early stage and homogenous HCC patient cohort with regular follow-up might explain the lower recurrence rate and early-stage recurrence pattern in this study. In addition, 63.8% of patients underwent antiviral therapy pre- or post-operatively, which might also reduce HCC recurrence post-curative resection^19,20^. However, two patients with recurrences in BCLC stage C, including one intrahepatic recurrence with portal branch invasion and the other with extrahepatic metastasis without intrahepatic recurrence, seemed inevitable with current guidelines of optimal surveillance interval and timely recall policies, which might be explained by aggressive tumor behavior^10,21^.

HCC recurrence is generally classified into early (within 1 or 2 years after curative resection) and late recurrence. While early recurrence is considered as resulting from intrahepatic metastasis by aggressive tumor behavior, late recurrence is de novo HCCs mainly owing to liver fibrosis background^10^. The predicting factors are tumor factors including tumor diameter, number, histology grade and microvascular invasion, and non-tumor factors including stage of liver disease. While liver stiffness was the only independent factor associated with recurrence after curative resection in this prospective cohort of patients, tumor factors were not. In this study, we enrolled patients with early stage HCC after curative resection and yielded a 1-year recurrence rate of 19.2%, lower than the 31% in a previous report^18^. The risk of intrahepatic metastasis might be low for this patient cohort with all early stage HCC and low histology grade, which explained low early recurrence rate after curative resection and no tumor factors associated with recurrence. However, liver advanced fibrosis or cirrhosis provided a background with higher hepatocarcinogenetic potential and resulted in higher development and recurrence post-curative treatment of HCC^4–6,18^. The incidence of de novo recurrence might continuously occur from early postoperative period until late period after resection^18^. In correlation with histological fibrosis stage, LS measured preoperatively was the independent factor in predicting all and late recurrences in this study. Our finding was compatible with that of the study from the same area^14^ and different from the recent study result in which spleen stiffness was the only predictor of late recurrence, instead of LS^16^. In contrast to the studies enrolling not only patients in heterogenous stage but also with macrovascular invasions^14,16^, our study clarified this issue with a homogenous and early stage HCC cohort in which curative resections were indicated in the guidelines^4–6^. Therefore, our result is convincing and useful for HCC patients for whom curative resection was performed according to current recommendations.

Although LS was useful in the prediction of HCC recurrence after resection, the performance and validities were not satisfactory in clinical practice. The prediction performance was 0.641 being similar to that in a previous study^14^. The optimal threshold in predicting recurrence varied for differences in the study populations. Instead of 13.4 kPa proposed in a previous study^14^, the optimal cutoff was 8.5 kPa, which might be owing to a lower proportion of patients in advanced fibrosis and cirrhosis stages (64.8% vs 81.2%) in this study cohort. However, both studies showed similar recurrence curves in demonstrating significant difference in recurrence after 1-year post resection stratified by 8.5 kPa and 13.4 kPa respectively. In addition to LS, insulin resistance by HOMA-IR was the independent factor associated with recurrence after 1-year post resection. Hyperinsulinemia has been considered to be involved in the progression and recurrence of HCC owing to the mitogenic and proliferative effects of insulin^22^. Insulin resistance has been identified as a risk factor of HCC development in chronic hepatitis C^22,23^. This study showed that the optimal cutoff of HOMA-IR index was 2.3, which was the same value proposed by the other study enrolling patients after curative treatments^24^.

Similar to other studies^23,24^, metabolic abnormalities including metabolic syndrome, diabetes, body mass index, waist and hyperlipidemia were not associated with recurrence in our study. We also demonstrated that combined HOMA > 2.3 and LS > 8.5 kPa identified those patients with high risk of HCC recurrence after 1-year post resection. Based on our study result, it might be beneficial for patients to improve insulin resistance and LS with life style changes and antiviral treatment in suppressing HCC recurrence. However, the risk prediction of HCC recurrence might be further improved with developments of risk scores or other models^25,26^.

There were some limitations in this study. Transient elastography with M-probe was used for LS measurement, which might result in higher LS for patients with body mass index more than 30 kg/m^2^^27^. Despite little effect on LS measurement due to small tumor diameter in our study, there might be over- or under-estimation of LS for patients with tumors located in the right liver. Spleen stiffness was not measured in this study. Most patients (92.6%) in this study were patients with chronic hepatitis B or C. Whether spleen stiffness and other etiologies were independent factors of HCC recurrence might need further study. For clinical practice, it might be necessary to validate the proposed cutoffs of LS and HOMA-IR in a large cohort study.

In summary, LS measured by transient elastography was the independent risk factor of HCC recurrence for patients with BCLC early stage HCC after curative resection. Insulin resistance by HOMA-IR was the independent factor for recurrence after 1-year post resection. With the cutoffs of 8.5 kPa and 2.3, LS and HOMA-IR stratified the risks of recurrence after 1-year post resection for early stage HCC after curative resection.

## Patients and methods

### Patients

This study was approved by the Institutional Review Board of Chang Gung Memorial Hospital (IRB number: 101-1075A3) and conducted in accordance with the 1975 Declaration of Helsinki. Consecutive patients fulfilling all inclusion and exclusion criteria were enrolled prospectively. Patients with suspicion of HCC who had undergone curative resection and Child–Pugh classification A liver function reserve signed informed consents. The inclusion criteria were patients with HCC in BCLC very early or early stage. The exclusion criteria were patients with history of hepatic malignancy or underwent two-stage resection. While the demographics and baseline clinical characteristics including metabolic syndrome, lipid profiles and homeostatic model assessment for insulin resistance index (HOMA-IR) were recorded before resection, the histological characteristics of tumors and surrounding normal hepatic parenchyma were recorded after resection. All patients were followed up regularly with imaging studies including hepatic ultrasonography, computed tomography and magnetic resonance imaging. The patients were followed up until tumor recurrence, death or end of 2018. All patients signed informed consents before enrollment.

### Liver stiffness measurement

LS was measured by using transient elastography (FibroScan, Echosens, Paris, France) with an M-probe before resection. It was performed in an overnight fasting state and by an experienced technician. The right lobe of the liver was assessed through the intercostal space while the patients were lying in a supine position with their right arms at maximal abduction. LS results were expressed as a median value with an interquartile range (IQR) in kilopascal (kPa)^28^. The results were considered reliable only when 10 successful shots, a successful rate more than 60%, and the IQR-to-liver stiffness ratio < 0.30 had been obtained.

### Statistical analysis

Quantitative variables were expressed with mean ± standard deviation or median with a range. Qualitative variables were expressed as absolute and relative frequencies. While Mann–Whitney U test and Student t test were used for comparisons of quantitative variables, Chi-square and Fisher’s exact tests were used in categorical variables. The correlations between liver stiffness values and histological fibrosis were analyzed with Spearman’s rank correlation method. Cox regression model was performed to determine independent factors associated with tumor recurrence. Hazard ratios (HR) and corresponding 95% confidence intervals (CI) were indicated^29^. The diagnostic performance of independent factor in predicting tumor recurrence was evaluated by receiver operating characteristic (ROC) curve. The area under the ROC curve (AUROC) and the 95% confidence interval were used as indexes of accuracy. The optimal cutoff values were determined with Youden index from ROC curve^30^. All data were recorded and analyzed using the SPSS v18 software package (SPSS Inc, Chicago, IL, USA); all p values were derived from 2-tailed tests, and a level of < 0.05 was accepted as statistically significant.
